# Microenvironment-Cell Nucleus Relationship in the Context of Oxidative Stress

**DOI:** 10.3389/fcell.2018.00023

**Published:** 2018-03-09

**Authors:** Shirisha Chittiboyina, Yunfeng Bai, Sophie A. Lelièvre

**Affiliations:** ^1^Department of Basic Medical Sciences, Purdue University, West Lafayette, IN, United States; ^2^3D Cell Culture Core, Birck Nanotechnology Center, Purdue University Discovery Park, West Lafayette, IN, United States; ^3^Center for Cancer Research, West Lafayette, IN, United States

**Keywords:** reactive oxygen species, chromatin, epigenome, tissue stiffness, aging, cancer, neurodegenerative disorders, stem cell

## Abstract

The microenvironment is a source of reactive oxygen species (ROS) that influence cell phenotype and tissue homeostasis. The impact of ROS on redox pathways as well as directly on epigenetic mechanisms and the DNA illustrate communication with the cell nucleus. Changes in gene transcription related to redox conditions also influence the content and structure of the extracellular matrix. However, the importance of microenvironmental ROS for normal progression through life and disease development still needs to be thoroughly understood. We illustrate how different ROS concentration levels trigger various intracellular pathways linked to nuclear functions and determine processes necessary for the differentiation of stem cells. The abnormal predominance of ROS that leads to oxidative stress is emphasized in light of its impact on aging and diseases related to aging. These phenomena are discussed in the context of the possible contribution of extracellular ROS via direct diffusion into cells responsible for organ function, but also via an impact on stromal cells that triggers extracellular modifications and influences mechanotransduction. Finally, we argue that organs-on-a-chip with controlled microenvironmental conditions can help thoroughly grasp whether ROS production is readily a cause or a consequence of certain disorders, and better understand the concentration levels of extracellular ROS that are necessary to induce a switch in phenotype.

## Introduction

The generation of reactive oxygen species (ROS) is part of normal physiology. Overproduction of ROS or insufficient enzymatic conversion of these molecules via antioxidant mechanisms results in oxidative stress that contributes to aging and disease. Oxidative phosphorylation, which provides cellular energy, is at the heart of ROS generation in the mitochondria, since it also results in the formation of superoxide anion (${\text{O}}_{2}^{-}$), hydroxyl radical (OH^.^) and hydrogen peroxide (H_2_O_2_) (Murphy, 2009); normally, ROS-induced translocation of signal transduction proteins and transcription factors to the nucleus promotes the expression of protective antioxidant enzymes (Poon and Jans, 2005; Kodiha et al., 2010). Other causes of intracellular ROS production include responses to infection, mental stress, physical exercise, and aging (Powers and Jackson, 2008; Bouayed et al., 2009; Romano et al., 2010; Ivanov et al., 2017). Interestingly, the generation of ROS in response to bacterial infections alters the host metabolism, triggering inflammatory signaling pathways that affect the transcription of proinflammatory and procarcinogenic genes such as cyclooxygenase 2, which may lead to metabolic diseases (Cassell, 1998; Mager, 2006; Ivanov et al., 2017), hence linking infectious disease to chronic disorders via ROS. The microenvironmental origin of ROS is due to extravasation and the activity of extracellular catalase, superoxide dismutase (SOD) and NADPH oxidases (NOX) in response to food and alcohol consumption and to pollutants such as heavy metals, cigarette smoke, and radiation (Limòn-Pocheco and Gonsebatt, 2009; Bauer et al., 2014). Importantly, although extracellular H_2_O_2_ easily enters in the cells (Ohno and Gallin, 1985; Limòn-Pocheco and Gonsebatt, 2009), thus potentially adding to intracellular burden in case of oxidative stress, its implication in health homeostasis remains poorly understood.

Theoretically, there are at least two means for microenvironmental ROS to affect cellular homeostasis via an impact on the cell nucleus. Direct diffusion of H_2_O_2_ into cells might contribute to high intracellular ROS that has been linked with alterations in chromatin organization and gene transcription (Rahman, 2002; Sundar et al., 2013; Kreuz and Fischle, 2016). Moreover, ROS-mediated activation of fibroblasts in the extracellular matrix (ECM) increases collagen I production (Tanaka et al., 1993), hence modifying tissue stiffness, which might influence gene expression in neighboring cells via mechanotransduction (Humphrey et al., 2014; Mouw et al., 2014; Handorf et al., 2015).

The nuclear compartment possesses an exquisite organization of chromatin necessary to maintain cellular homeostasis via its impact on the epigenome (Abad et al., 2007; Lelièvre, 2009; Grummt, 2013). Oxidative stress can be specifically sensed by cell nuclei (Provost et al., 2010), in part due to mitochondrial stress leading to signal transduction and the nuclear accumulation of respiratory enzymes like CDC like kinase 1 (CLK1). Specifically, mitochondria-nucleus cross talk controls the response to oxidative stress. For instance, the expression of DNA methyl transferase 1 (DNMT1) that mediates epigenetic changes in mitochondria is controlled by transcription factors responsive to oxidative stress (Shock et al., 2011), and nuclear CLK1 maintains mitochondrial homeostasis by regulating genes in the cell nucleus that deplete ROS (Monaghan et al., 2015). H_2_O_2_ entering cells can be metabolized into OH^.^ known to induce DNA lesions (Tsunoda et al., 2010); but the strong cellular influence of ROS might not involve such damage (Kirkland, 1991), suggesting that beneficial and deleterious effects of ROS likely involve transcriptional effects.

Here, we discuss how extracellular ROS might contribute to normal aging and diseases via a dual influence on the microenvironment, notably tissue stiffness, and on cellular homeostasis. Knowledge on chemical and physical consequences of incremental ROS on the cell nucleus is presented before proposing new *in vitro* models to help fill the gaps to understand the determining impact of ROS thresholds.

## Reactive oxygen species and cellular homeostasis

### A fine line between normal and abnormal stem cell differentiation

High levels of ROS damage macromolecules, yet ROS is necessary for normal biological processes (Schieber and Chandel, 2014). Embryonic stem cell differentiation requires increased ROS and ATP production in mitochondria, as shown for the cardiovascular tissue (Schmelter et al., 2006). There is also upregulation of NOX within cells and the microenvironment. Yet, additional intracellular ROS, due to entry of environmental H_2_O_2_, might inhibit nuclear translocation of proteins responsible for the antioxidant response by binding to their cysteine motifs (Lennicke et al., 2015). Indeed, oxidative stress has been reported to impair the proliferation of embryonic stem cells (Brandl et al., 2011), but whether abnormally high microenvironmental ROS during embryogenesis alters organ development remains to be clearly determined.

**Figure 1:**
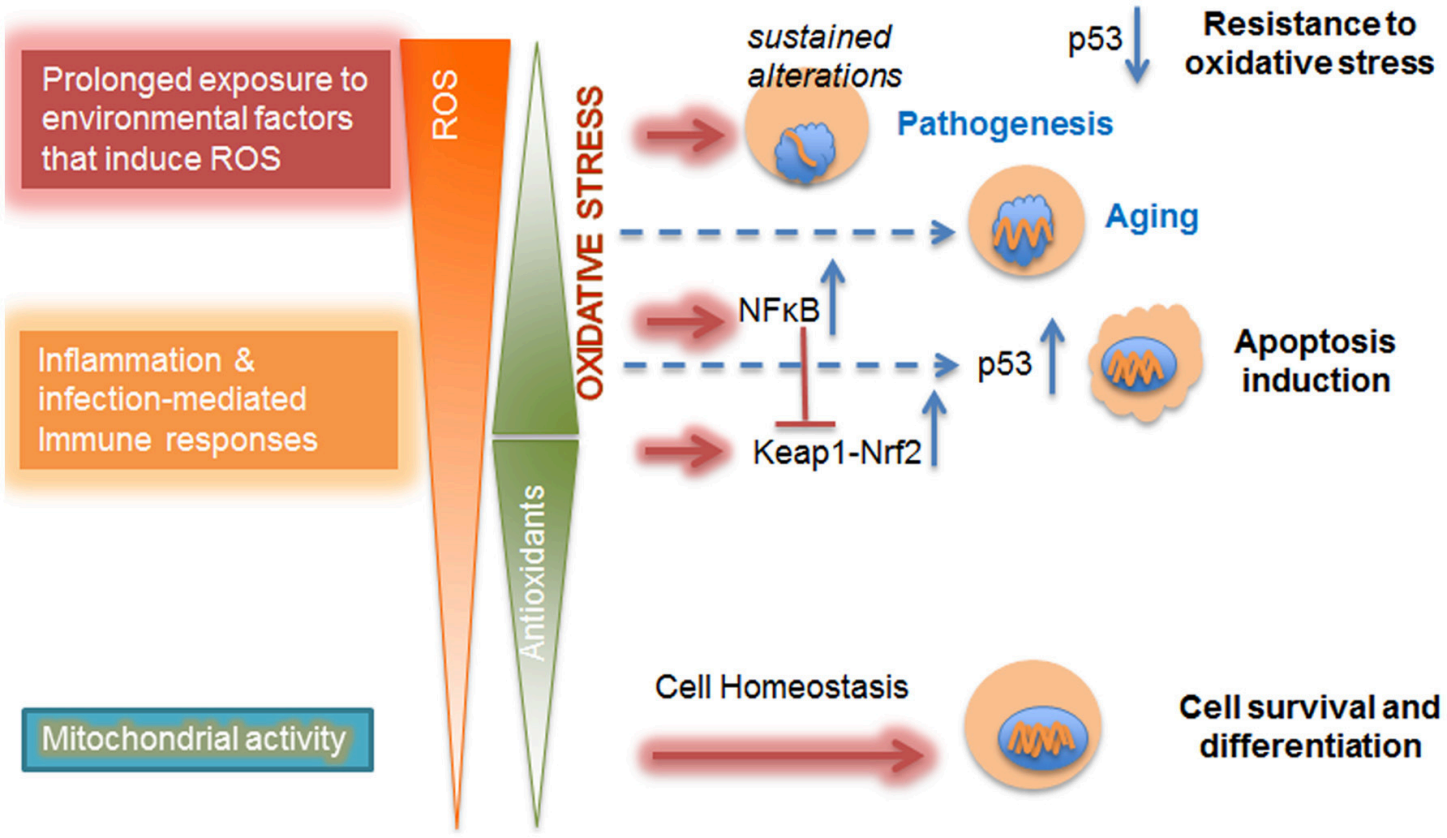
Dose-dependent impact of ROS on cellular metabolism. Mitochondrial activities, such as oxidative phosphorylation, contribute to physiological levels of ROS that are counterbalanced and detoxified by antioxidant defense mechanisms. These ROS are produced as a response to increased cellular demand for energy and are essential for cell survival, differentiation, and tissue development. With the increase in imbalance between ROS and antioxidant levels due to inflammation or prolonged exposure to environmental factors, there is a shift in redox regulation pathways from Keap-Nrf2 to NFκB. At mild oxidative stress level p53-mediated cell death (apoptosis) is observed. Further increase in oxidative stress level in diseased cells inhibits p53-induced cell apoptosis and promotes resistance to oxidative stress. Furthermore, chronic oxidative stress leads to altered gene expression and changes in nuclear morphology already observed in aging; the level at which excess ROS might contribute to sustained alterations in the epigenome that trigger pathogenesis might depend on microenvironmental conditions (Chittiboyina et al., 2018). Nuclei are shown in blue and increasing alterations in the nucleus are displayed as shortening orange wiggles.

The balance of self-renewal and cell-type specific differentiation, two functions controlled by low levels of ROS, is essential for the maintenance of a stem cell pool within adult organs (Maraldi et al., 2015; Cieślar-Pobuda et al., 2017), with a fine line between desired stimulation and unwanted damage. Adult stem cell differentiation in the central nervous system is directed by lens epithelial-derived growth factor (LEDGF), itself involved in the protective response to oxidative stress (Chylack et al., 2004; Basu et al., 2016). Stem cells have defective DNA repair capacity, which is further exacerbated by ROS (Cieślar-Pobuda et al., 2017). Prolonged exposure to ROS has been shown to result in cell senescence *in vitro* (Kuilman et al., 2010; Davalli et al., 2016) and has been proposed to contribute to pathologies associated with aging such as cancer and Alzheimer's disease in a dose-dependent manner (Sarsour et al., 2009; Zhu et al., 2013; Childs et al., 2015; Sikora et al., 2015; Qiu et al., 2017) (Figure 1).

For instance, stem cell self-renewal and resulting premature pool exhaustion occurs with a moderate increase of ROS concentration (Zhou et al., 2014; Maraldi et al., 2015). Understandably, detrimental exposure to ROS has to be chronic when at low dose, and, *in vitro*, it seems to preferably trigger the activation of the p38-p16 pathway that induces stem cell senescence (characterized by loss of replicative capability) (Shao et al., 2011); whereas acute and high ROS dose exposure activates the p53 pathway by accumulation of ataxia telangiectasia mutant (ATM) kinase in the cell nucleus, triggering not only stem cell aging (characterized by a diminished capacity to function and respond to the microenvironment), but also apoptosis (Mai et al., 2010; Liu and Xu, 2011).

The threshold at which an imbalance of ROS and thus, oxidative stress, leads to pathologies linked to an effect on stem cells might be low (Bigarella et al., 2014). The contributions to such threshold of extracellular ROS coming from the degradation of our environment and dietary habits remain unanswered questions.

### Oxidative stress in the normal process of aging

Aging is the major cause of increased susceptibility to neurodegenerative diseases, cancer, and other metabolic disorders. It has been characterized as a progressive loss of tissue functions due to cumulative damages in cells and their microenvironment. The original free-radical theory of aging and derived mitochondrial theory of aging consider ROS as the main cause for these damages. Indeed, oxidative stress has been connected with all of the nine potential hallmarks of aging (López-Otín et al., 2013), including genomic instability (Hoeijmakers, 2009), telomere attrition (Sun et al., 2015), epigenetic alterations (Guillaumet-Adkins et al., 2017), loss of proteostasis (Bader and Grune, 2006), deregulated nutrient sensing (Luo et al., 2017), mitochondrial dysfunction (Harman, 1965), cellular senescence (Davalli et al., 2016), stem cell exhaustion (Cieślar-Pobuda et al., 2017), and altered intercellular communication (Poli et al., 2004). The unpaired electron of the active oxygen molecule of ROS can capture another electron from macromolecules such as DNA, lipids, and proteins resulting in changes in biological properties; however, the molecular alterations necessary for aging are still poorly understood. Furthermore, the free-radical theory of aging is challenged by the mixed results of gene knockout studies in animal models showing that on one hand, lifespan can be expanded by decreasing ROS level, and on the other hand, increasing ROS level has no effect or might even prolong the lifespan in individual mice (Hamilton et al., 2012). Among studies related to ROS and performed in mice, mutation and knock-out models have been developed that impair transcription factors TP53INP1, JunD, ATM, Forkhead box O (FOXO) and p53 normally involved in tumor suppression (Sablina et al., 2005; Reliene and Schiestl, 2006; Tothova et al., 2007; Laurent et al., 2008; Cano et al., 2009). As a result, there is an increased ROS level in mice suggesting that these transcription factors play a role in antioxidant defense. Moreover, an association between ROS accumulation and specific aging characteristics has been reported in multiple types of adult stem cells. For instance, ROS level increases in murine hematopoietic stem cells when FOXO is knocked down, which results in stem cell self-renewal exhaustion (Miyamoto et al., 2007; Tothova et al., 2007). It remains largely elusive whether and how deficiency of FOXO family members can lead to aging-related diseases; however, Genome Wide Association Studies conducted in human population samples revealed a positive link between FOXO genes and extreme longevity (Martins et al., 2016). Among other examples of comparable murine and stem cell studies, a genetic knockout of deacetylase Sirtuin family members in mice (Mohrin et al., 2015) as well as in neural progenitor cells (Prozorovski et al., 2008) revealed the important function of sirtuins in ROS balance and stem cell aging.

The aging process might rely on repair ability, especially for DNA, more than the ROS level in the organism (Lewis et al., 2013; MacRae et al., 2015). Repair ability has been linked to epigenetic pathways (Dinant et al., 2008; Lahtz et al., 2013; Montenegro et al., 2016), and it is controlled by the ECM that influences proteins involved in higher order chromatin organization (Vidi et al., 2012). Microenvironmental ROS can damage the ECM and cell surface proteins, leading to altered cell adhesion and signaling like in atherosclerosis, a disease of aging (Kennett et al., 2011). Aging in general has been associated with an initial increase followed by a decrease in ECM stiffness (Achterberg, 2014), and tissue stiffness depends on the impact of ROS on fibroblasts (Tanaka et al., 1993; Siwik et al., 2001; Lijnen et al., 2012). Therefore, understanding the relative contribution of ROS to aging in different organs should take into account the microenvironment.

### Oxidative stress in disease development and progression

In cells with high energy requirement the microenvironment contributes to oxidative stress. In normal cells, this is a concern with embryonic development as discussed above. In diseases, concerns are with cells associated with a metabolic syndrome (e.g., cardiovascular disease, diabetes) and other cells with high metabolic demands like in cancers and neurodegenerative disorders. Cell-required increased energy production, and thus ROS, by mitochondria is fulfilled by extracellular growth factors and hormones (Turpaev, 2002; Ward and Thompson, 2012).

The microenvironment also participates in ROS-mediated impact in disease via stromal cells. Under oxidative stress these cells secrete lactate and pyruvate, providing an alternate source of energy called “aerobic glycolysis” (or Warburg effect) in rapidly proliferating cancer cells (Gatenby and Gillies, 2004; Liberti and Locasale, 2016). Extracellular ROS activate stromal cells (Lijnen et al., 2012). Activated fibroblasts lose caveolin 1, which has been associated with poor survival in patients with triple negative breast cancer and with early breast cancer recurrence in general (Witkiewicz et al., 2009, 2010; Popovska et al., 2014). They also increase collagen I secretion, resulting in tissue stiffness (Karsdal et al., 2013; Eble and de Rezende, 2014), a condition associated with the aggressiveness of certain cancers (Hoyt et al., 2008; Acerbi et al., 2015; Northey et al., 2017). Whether changes in tissue stiffness associated with cancer progression truly result from oxidative stress remains to be confirmed.

Brain susceptibility to oxidative stress is in part linked to low levels of antioxidant mechanisms in the microenvironment leading to high amounts of remaining ROS (Uttara et al., 2009). An oxidative microenvironment is a feature of Alzheimer's disease that promotes the production and aggregation of extracellular amyloid β plaques by influencing the activity of α- and β-secretases (Behl, 2005; Mosconi et al., 2008). Moreover, cytoplasmic plaque accumulation triggers the overproduction of intraneuronal ROS by disturbing mitochondrial activity (Xie et al., 2013). In Parkinson's disease microenvironmental oxidative stress is due to the production of ${\text{O}}_{2}^{-}$ triggered by microglial cells, with immediate conversion to H_2_O_2_ species that attack the surrounding neurons, eventually leading to neurodegeneration (Dias et al., 2013).

Chronic diseases like cancer and neurodegenerative disorders, with a microenvironmental component to their onset and progression, are linked to alterations in the epigenome, but deciphering the contribution of oxidative stress to epigenetic alterations is a difficult task.

## Incremental impact of ROS on the cell nucleus

A major recipient of ECM signaling, the cell nucleus reflects cell phenotypes (Bissell, 1981; Lelièvre, 2010). Changes in the epigenome (i.e., the collection of epigenetic marks that control transcription profiles) and morphometry (notably size and shape) of the nucleus accompany differentiation disorders, including cancer and neurodegeneration (Zink et al., 2004; Lelièvre, 2009; Lattanzi et al., 2012). Beyond oxidative DNA damage induced by hydroxyl radicals that is not necessarily associated with disease development (Evans et al., 2004; Silva et al., 2014; Pereira et al., 2016), ROS might influence transcription depending on their concentration and origin.

### Impact of ROS on protein activation and translocation to the cell nucleus

Transcription regulation mediated by ROS occurs already in the nanomolar range of H_2_O_2_ (Schieber and Chandel, 2014) acting as redox signaling, mainly through reversible thiol modifications on phosphatase and kinase cysteine residues (Janssen-Heininger et al., 2008; Collins et al., 2012). Orchestrated enhancement of tyrosine kinase (Paulsen et al., 2011; Heppner et al., 2018) and inhibition of tyrosine phosphatases (Sundaresan et al., 1995; Bae et al., 1997; Denu and Dixon, 1998; Lee and Esselman, 2002) by H_2_O_2_ amplify the activation of PI3K/AKT and transcription mediators (e.g., STAT), that favor cell proliferation and survival. Cysteine modifications by low H_2_O_2_ concentrations are likely to maintain the expression of stress-responsive genes at basal level under normal conditions. This pathway involves Kelch-like ECH-associated protein 1 (Keap1) in which H_2_O_2_ modifies cysteines leading to the release and translocation of nuclear E2-factor-related factor 2 (Nrf2) into the nucleus, where it binds antioxidant response elements in the promoter of detoxification genes (McMahon et al., 2003), notably in response to inflammation (Suzuki and Yamamoto, 2017). This pathway seems attenuated in neurodegenerative diseases (Gan and Johnson, 2014; Buendia et al., 2016), whereas constitutive activation is commonly observed in cancers and linked to cell survival and resistance to ROS production-based therapies (Leinonen et al., 2014).

Concentration dependent regulation of signaling pathways by ROS is essential for tissue survival. Physiological levels of ROS are known to upregulate and activate proinflammatory cytokines such as IL-6 and IL-4 (Frossi et al., 2003), whereas elevated ROS can lead to activation of IL-6 that mediates Nrf2 translocation to the nucleus and subsequent upregulation of antioxidant mechanisms (Theodore et al., 2008; Hsieh et al., 2009). Cytokines are also linked to the NF-κB/p53 pathway under ROS stimulation. Tumor suppressor and transcription factor p53 participates in redox-responsive control of the cellular stress response (Budanov, 2014). Although NF-κB and p53 have opposing effects on cellular apoptosis, both are activated and translocate to the cell nucleus under ROS, and regulate the transcription of IL-6 (Lowe et al., 2014). Interestingly, p53 is shown to suppress Nrf2-mediated antioxidant response, but not the expression or activation of Nrf2 itself in mouse hepatocarcinoma cells (Faraonio et al., 2006), hence these two proteins seem independent from each other regarding their involvement with IL-6 pathway. Low levels of ROS do not induce NF-κB activation and nuclear translocation. Yet, if ROS levels lead to oxidative stress, NF-κB becomes activated and promotes cytokine-mediated inflammatory pathways. However, tissue insults from proinflammatory cytokines are countered by high levels of ROS that upregulate and activate anti-inflammatory cytokines such as IL-10 (Kelly et al., 2010; Latorre et al., 2014). If further strengthened, oxidative stress may trigger p53-mediated apoptosis and even inhibit p53 activity via oxidation of certain cysteine residues, which prevents the antioxidant response in cells, leading to further accumulation of ROS (Cobbs et al., 2001; Bensaad and Vousden, 2005; Halliwell, 2007).

At least part of H_2_O_2_ involved in low level activation is transiently produced by the oxidation of cell membrane-bound NOXs that are recruited by receptor binding of extracellular growth factors and hormones (Sundaresan et al., 1995). The involvement of such microenvironment-mediated cytoplasmic production of ROS in detrimental levels of oxidative stress remains to be determined.

### Impact of ROS on epigenetic mechanisms

In cancer, diabetes and Alzheimer's disease, alterations in epigenetic pathways have been linked to oxidative stress, although the concentrations of ROS that matter are unknown. Epigenetic pathways encompass DNA methylation and several posttranslational modifications (methylation, acetylation, phosphorylation, ubiquitination) on various histone residues (Kreuz and Fischle, 2016; Guillaumet-Adkins et al., 2017). The combination of some of these epigenetic traits determines the level of transcription of a particular gene. As shown in the selected examples below, all of these types of epigenetic modifications can be affected by ROS.

ROS-induced global heterochromatin loss may follow DNA damage that, in turn, promotes chromatin relaxation (Pal and Tyler, 2016), and might be linked to reduced S-Adenosyl Methionine synthesis, caused by oxidized methyl adenosine transferase (Towbin et al., 2012). Direct oxidation of 5 methyl cytosine (5-mC) by ROS might also inhibit DNMT1, contributing to demethylation of CpG sites on the DNA. In contrast, DNA hypermethylation might be due to oxidative stress-mediated inhibition of methyltransferase-related TET proteins leading to an increase in 5-mC level (Chia et al., 2011; Wu and Zhang, 2017). Pericentromeric heterochromatin stimulated by oxidative stress is associated with increased expression of SIRT1 that stabilizes SUV39H1, leading to increased histone H3 trimethylated on lysine 9 (H3K9me3) (Bosch-Presegué et al., 2011).

Under oxidative stress histone demethylases are inhibited leading to H3K9me2/3 and H3K27me3 increase (Chervona and Costa, 2012; Niu et al., 2015; Kreuz and Fischle, 2016), which may repress transcription. Acetylation of histone lysine residues associated with chromatin relaxation and transcriptional activation might also occur following inhibition of histone deacetylases (Ropero and Esteller, 2007). Notably, ROS increases acetylated histone 4 (Tomita et al., 2003) and histone 3 (Choudhury et al., 2010). Several studies have shown histone phosphorylaton under oxidative stress, for example H2AX and histone 3 (Katsube et al., 2014; Marwick et al., 2015). Chronic ROS leads to H2AX poly-ubiquitination due to increased interaction between H2AX and E3 ubiquitin ligase RNF168, which results in reduced level of H2AX and increased sensitivity of cancer cells to chemotherapy (Gruosso et al., 2016). Thus, depending on the posttranslational modifications the resulting impact might be on the regulation of gene expression, DNA replication and DNA repair, making ROS a key regulator of all these processes via epigenetic influence.

Most interestingly, extracellular ROS alter nuclear morphometry (e.g., size and shape) (Barascu et al., 2012), in part by disrupting lamins involved in the structural organization of the nucleus (Shimi and Goldman, 2014). Peripheral heterochromatin is organized into lamina-associated domains that directly participate in the control of heterochromatin linked with the degree of tissue differentiation (Gonsalvez-Sandoval et al., 2013; Solovei et al., 2013). Impaired interaction between nuclear envelope and heterochromatin proteins, like HP1, leads to the mislocalization of heterochromatin (Eskeland et al., 2007; Schneider and Grosschedi, 2007), which could affect transcriptional regulation. We have shown that nuclear morphometry responds to ROS in a dose dependent manner (Chittiboyina et al., 2018). The impact of oxidative stress-mediated alterations of nuclear morphometry on chromatin organization and gene transcription remains to be studied.

Epigenetic changes might be protective against DNA damage and mediate ROS resistance in cancer. Many contributing experiments included extracellular H_2_O_2_, which calls for clarifications regarding the contribution of microenvironmental ROS in these nuclear events.

## Improving knowledge on the role of oxidative stress in cellular homeostasis

The antioxidant cellular mechanisms activated in response to elevated levels of ROS are fairly known. Glutathione (GSH) can directly scavenge super oxide anions (O2^−.^), one of the most reactive free radicals, while GSH peroxidases, peroxiredoxins and thioredoxins all target H_2_O_2_ (Hanschmann et al., 2013). The expression of these antioxidant enzymes is due to the activation and resulting cytoplasmic to nuclear translocation of transcriptional coactivators Nrf2 and LEDGF upon ROS generation (Sharma et al., 2000; McMahon et al., 2003). However, the mechanisms by which these transcriptional coactivators sense altered ROS homeostasis and may themselves be compromised, leading for instance to oxidative stress resistance in cancer (Freitas et al., 2012; Balvan et al., 2015), remain to be understood.

Elevated ROS levels have been reported in various pathologies that result from epigenetic alterations and for which microenvironmental modifications are instrumental. For instance, oxidative stress leads to increased ECM stiffness (Eble and de Rezende, 2014). Enhanced microenvironmental stiffness triggers mechanotransduction via stimulation of the cytoplasmic form of transcriptional activator Yes-associated protein (YAP) (Dupont et al., 2011), leading to its relocalization to the cell nucleus. Upon reaching chromatin YAP may activate the transcription of genes such as those coding for β-catenin, ErbB4, FoxO1 that are involved in apoptosis, cancer progression and stem cell self-renewal (Dupont et al., 2011; Zhu et al., 2015; Moleirinho et al., 2017). Interestingly, ECM stiffness may not only activate YAP, but also regulate its transcription (Low et al., 2014). The modulation of mechanical strength affects the actin cytoskeleton, which in turn, influences YAP phosphorylation and thus, its nuclear localization (Das et al., 2016). Hence, mechanotransduction generated by changes in ECM stiffness has posttranslational and transcriptional effects. How the combined actions of microenvironmental ROS on ECM composition (via an effect on fibroblasts) and directly on epithelial cells (via chromatin regulation, as discussed in previous sections), ultimately transforms the cell phenotype is difficult to study in simple cell culture as well as in complex whole organisms like in mice. Thus, the mechanisms of microenvironmental ROS-induced differentiation and pathogenesis remain to be clarified. Moreover, the dual impact of ROS, either beneficial or detrimental, underlines the need to identify thresholds for action within the cell nucleus.

Microenvironmental impact on tissues is best studied with controlled 3D cell culture models (Lelièvre et al., 2017). Using a microfluidic system in which a gradient of H_2_O_2_ was delivered inside the ECM, we showed that there were thresholds for phenotypic response measured by nuclear morphometry depending not only on the tumor grade, but also on matrix stiffness (Chittiboyina et al., 2018). Microscale optics was used to assess H_2_O_2_ concentrations delivered in different regions of the culture chamber. However, this method is cumbersome and cannot be used in real time. Assessment of ROS impact on the cell nucleus was measured by indirect means, such as the expression of antioxidant genes, but there was no direct measurement of ROS concentration inside cells.

To better understand the relationship between cell nucleus and microenvironmental ROS production extracellular H_2_O_2_ should be measured. ROS concentration is not uniform, but rather a dynamic gradient as seen *in vivo* (Ogasawara and Zhang, 2009; Zorov et al., 2014). Measurements might be accomplished by placing biosensors within the ECM (Hynes et al., 2014). Once H_2_O_2_ enters inside cells, it remains there due to differences in diffusion radii (Huang et al., 2017). Intracellular ROS assessment relies on indirect and low precision methods such as antioxidant capacity or redox potential (Barzegar and Moosavi-Movahedi, 2011), using fluorescent probes that react with free radicals. To determine the contribution of microenvironmental ROS to intracellular ROS, it might be possible to use differential assessment techniques where mitochondrial ROS concentrations (primarily H_2_O_2_), detected using Mitotracker, are deducted from the total intracellular ROS. Noticeably, the nucleus is in a higher oxidizing state than mitochondria (Go and Jones, 2008), and maintenance of nuclear redox homeostasis is essential for proper transcription of antioxidants in response to oxidative stress (Provost et al., 2010; Espinosa-Diez et al., 2015). The nuclear accumulation of ROS may be measured by detection of Mn-Superoxide dismutase, but this enzyme is not influenced only by H_2_O_2_ (Miao and St. Clair, 2009; Wedgwood et al., 2011; Candas and Li, 2014). Nanoprobes might enable direct measurement of ROS in the nucleus, which would require removing interference from mitochondrial ROS possibly using Mitotracker (Puleston, 2015). Systems that track the production of ROS in the microenvironment and their transport to the nucleus through the cytoplasm are awaited since methods used to sense ROS in lower organisms with heavy metal and electrochemical probes or semi-stable paramagnetic compounds (Suárez et al., 2013; Koman et al., 2016) are considered too invasive and potentially toxic for mammalian cells.

## Conclusion

Due to their dual effect as a benefactor and also a damage inducer, ROS are re-emerging as therapeutic targets. Most ROS inducing therapeutics are effective only under a highly oxidative microenvironment, further supporting the role of the microenvironment in disease and therapy. As an example, the tissue damage induced by ROS is considered to be an advantage in order to target tumor cells using agents such as procarbazine that releases azo compounds in the highly oxidative environment of cancers (Vallejo et al., 2017). These compounds further generate ROS that target tumor cells. Unfortunately, such an increase in ROS has been linked to differentiation of cancer stem cells and rapid recurrence of cancer (Ding et al., 2015). Improved cancer therapy based on ROS might require a combination of ROS inducers and inhibitors of ROS-mediated stem cell differentiation pathways, requiring further understanding of the epigenetic mechanisms influenced by ROS.

**Figure 2:**
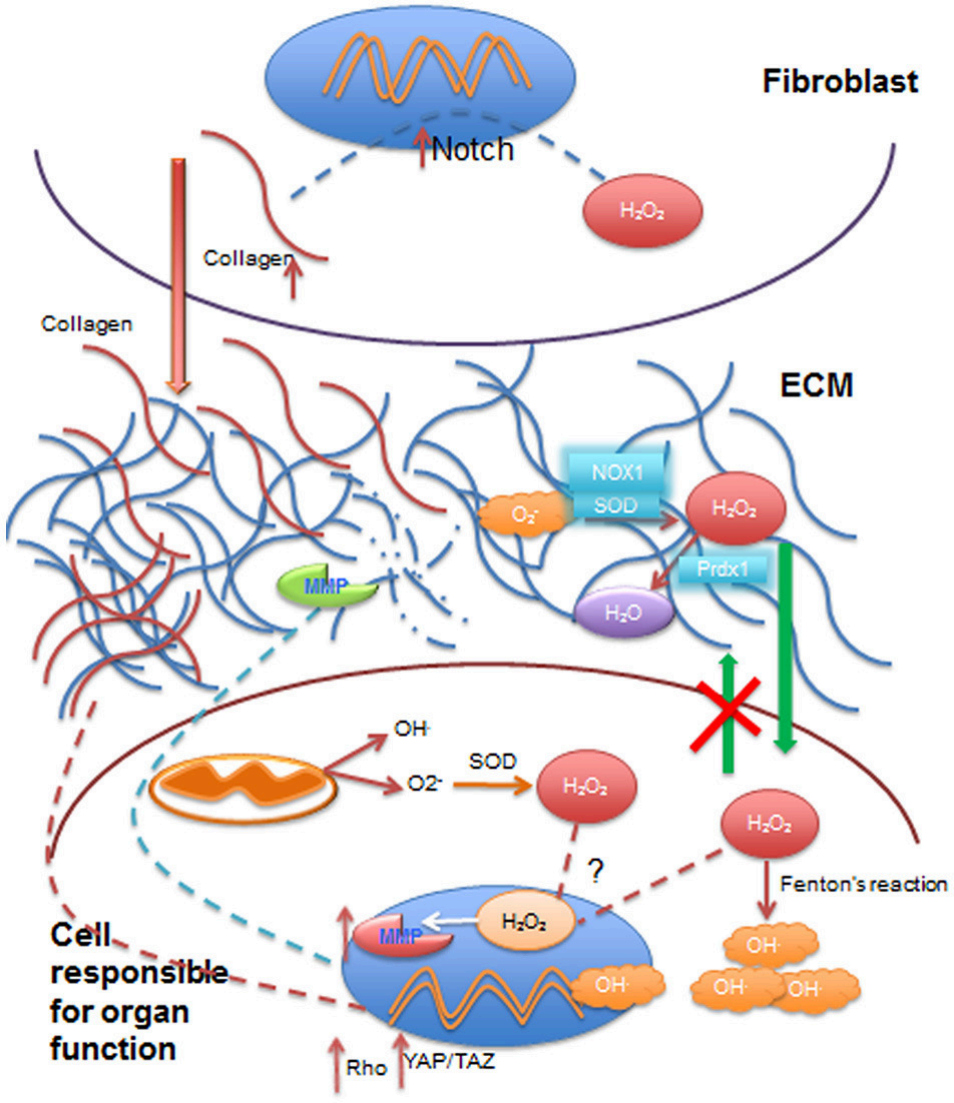
Summary of some effects of ROS on extracellular matrix, cytoplasm and cell nucleus involved in dynamic reciprocity. Reactive oxygen species (ROS), such as superoxide anion (O2^−^), transported from the vasculature to the extracellular matrix (ECM) are converted to hydrogen peroxide (H_2_O_2_) by superoxide dismutase (SOD) and NADPH oxidases (NOX1) in the ECM. H_2_O_2_ may be reduced to water (H_2_O) by reductases such as peroxireductase (Prdx1) in the ECM. Extracellular H_2_O_2_ can diffuse through cell membrane into the cytoplasmic compartment, but it cannot exit cells (green arrows), where it contributes to the increase in intracellular ROS levels by production of hydroxyl radicals (OH^.^) by Fenton's reaction, or it can be transported to the nucleus to activate the transcription of matrix metalloproteases (MMP). Collagen can be broken down by MMP activity in the ECM. Besides extracellular ROS, mitochondrial activity also contributes to intracellular ROS, which can further add to H_2_O_2_ going to the cell nucleus. Stromal cells such as fibroblasts are activated by ROS (primarily H_2_O_2_), which increases collagen production via Notch signaling activation. Increased collagen deposited in the ECM (red arrow) contributes to increased stiffness of the ECM that, in turn, activates mechanotransduction pathways such as Rho and YAP/TAZ signaling with an impact on gene transcription. Nuclei are depicted in blue and mitochondria in dark orange.

There is little doubt that microenvironmental ROS contributes to aging and disease. The concentration threshold necessary to induce lasting effects (via an impact on the epigenome) is essential to determine. Thorough investigations of the microenvironment are paramount in light of the influence of ROS on stromal cells and on the ECM. Physical alterations of the ECM nourish the concept of dynamic reciprocity between nucleus and microenvironment (Bissell, 1981), by possibly influencing the epigenome via mechanotransduction, which would consequently alter the impact of ROS on genes. Nuclear reciprocity targeting the ECM is illustrated by the fact that certain ROS-responsive pathways may control the expression of metalloproteases (Daugaard et al., 2007; Sims et al., 2011) that contribute to aging and cancer progression (Figure 2). Another example of reciprocity are the (epi)genetic alterations in cancer cell genes that regulate energy producing pathways, requiring more absorption of soluble factors from the microenvironment. Interestingly, tissue heterogeneity is emerging as an engine for cancer progression (Lelièvre et al., 2014; Mohanty et al., 2014; Kang et al., 2015). The microenvironment is likely a source of heterogeneity (Kim and Zhang, 2016; Natrajan et al., 2016) depending on variable local concentrations of oxygen, hormones, and growth factors. The contribution of extracellular ROS to such heterogeneity is an interesting avenue of investigation.

## Author contributions

SC wrote several portions of the manuscript, designed the figures and participated in edits. YB wrote parts of the manuscript and participated in edits. SL wrote parts of the manuscript and edited the entire manuscript.

### Conflict of interest statement

A patent disclosure was submitted regarding the gradient-on-a-chip (Chittiboyina et al., 2018). The authors declare that the research was conducted in the absence of any commercial or financial relationships that could be construed as a potential conflict of interest.
